# Cross-sectional computed tomography assessment of exophthalmos in comparison to clinical measurement via Hertel exophthalmometry

**DOI:** 10.1038/s41598-022-16131-4

**Published:** 2022-07-13

**Authors:** A. Klingenstein, C. Samel, A. Garip-Kübler, C. Hintschich, U. G. Müller-Lisse

**Affiliations:** 1 Department of Ophthalmology, Ludwig-Maximilians-University, Klinikum der Universität München, Campus Innenstadt, Mathildenstraße 8, 80336 Munich, Germany; 2 Institute of Medical Statistics and Computational Biology, Faculty of Medicine, University of Cologne, Cologne, Germany; 3 Department of Radiology, Ludwig-Maximilians-University, Munich, Germany

**Keywords:** Eye diseases, Medical imaging, Computed tomography

## Abstract

To determine protrusion assessment via Hertel exophthalmometry in comparison to measurement on Computed Tomography (CT). Retrospective blinded comparison of exophthalmos measurements on axial CT with Hertel exophthalmometry measurements in 113 patients. Descriptive statistics, Pearson’s correlation, Kruskal–Wallis and Mann–Whitney-U test were employed for analysis. Mean difference of proptosis between both eyes was 2.4 (SD ± 2.0) mm in CT and 2.2 (SD ± 2.0) mm in Hertel measurements. Proptosis of 0–2 mm was present in 69 (61.1%), and > 2 mm in 42 (38.9%) patients in Hertel measurements (CT 64 (56.6%), and 49 (43.4%) patients). Pearson’s coefficient showed a correlation of 0.793 between both methods (*p* < 0.001). Accuracy of Hertel measurement depended significantly from the examiners’ experience (< 5 (group 1), 5–15 (2) and > 25 (3) years, *p* = 0.042, Kruskal–Wallis analysis; *p* = 0.086 group 1 vs. 2, *p* = 0.014 group 1 vs. 3, *p* = 0.688 group 2 vs. 3, Mann–Whitney-U-test), reflected by levels of Pearson’s coefficient (correlation of both methods 0.691 (group 1), 0.837 (2) and 0.831 (3), respectively, *p* = 0.01). Generally, Hertel exophthalmometry correlates well with CT measurements. Subgroup analysis confirmed a superior quality of Hertel measurements in favour of experienced examiners. Teaching of accurate Hertel exophthalmometry should be improved. Assessment of exophthalmos using standardized criteria should be implemented for imaging reports.

## Introduction

Exophthalmos is a frequent and important symptom in orbital consultation and may manifest either uni- or bilaterally. In proptosis measurement, the antero-posterior position of the globe relative to the orbital rim is assessed^1,2^. A wide spectrum of both benign and malignant diseases must be considered for differential diagnosis causing exophthalmos ranging from inflammatory, vascular, posttraumatic to neoplastic pathologies. Neoplastic malignancies underlying exophthalmos are benign in approximately 1/3 and malignant in 2/3 of cases with malignant tumors increasing rigorously with patient age^3^.

Clinical measurement of exophthalmos is commonly performed by Hertel exophthalmometry^1,4^. In this examination, the distance between both orbital rims (base) and the level of the lateral orbital rim to the corneal apex are assessed. The normal distance from the orbital rim to the corneal apex is 12–21 mm, with the upper limit of people of African origin being slightly higher (about 23–24 mm)^5^. Generally, a difference of > 2 mm between both sides is considered pathologic^6^. Inter- and intra-observer variability of this measurement must be considered as problematic^4,7–10^ and for reliable assessment, clinical experience is of importance. Furthermore, clinical measurement of exophthalmos may be challenging in cases with severe upper eyelid swelling, eyelid ptosis, vertical deviation of the globe or poor patient compliance^11^.

Radiologic exophthalmos measurements via CT have been reported in patients with suspected dysthyroid optic neuropathy^11,12^, but also for other orbital pathologies^13^ and have proven a high repeatability and reproducibility^11^. An additional advantage of radiographic evaluation of exophthalmos is permanent recording of the images (which may be especially helpful for longitudinal imaging comparison, e.g. before and after therapy or decompression surgery).

Different methods for assessment of exophthalmos on CT have been described previously, e.g. measuring the distance from both lateral rims to the corneal surface, the distance from the lateral to medial orbital rims to the corneal surface, both in the axial plane, respectively, or the distance of the corneal apex from the superior and inferior orbital rim on the sagittal plane^11^.

In the present study, we compared clinical measurement of exophthalmos via Hertel exophthalmometry to assessment on axial CT-imaging to evaluate differences in measurements of the base and protrusion of the globe. We hypothesized that clinical Hertel exophthalmometry measurements correlate with the level of experience of the examiners. Furthermore, subgroup correlation analysis of proptosis measurements and final diagnosis was performed.

## Material and methods

We retrospectively assessed exophthalmos on cross-sectional diagnostic CT-imaging of 113 patients who presented in the oculoplastic department of Ludwig-Maximilians-University, Munich, Germany and underwent imaging between 05/2012 and 02/2020 and whose imaging was performed in our department of Radiology within a median time difference of 4 (mean 15.7) days of clinical presentation.

The ethics-committee of Ludwig-Maximilians-University, Munich, Germany, decided that this retrospective single-center observational cohort-study in selected patients did not require ethical advice and waived individual patient consent (vote number 20-633-KB). However, written informed consent for MD (multi-detector) CT-work-up was obtained from all patients. All patient data is non-identifiable and complied with relevant data protection and privacy regulation. All methods were performed in accordance with the relevant guidelines and regulations.

Clinical data were collected from the original patient files. Hertel exophthalmometry was performed by either a resident in Ophthalmology (< 5 years of clinical experience, group 1), an Ophthalmologist specializing in oculoplastic surgery (5–15 years of clinical experience, group 2), or a consultant with more than 25 years of clinical experience (group 3), respectively. The clinical Hertel measurement was performed with the patient sitting upright and the patients’ head in the primary position and the examiners' eyes at the same level as the patients’ eyes in a well-lit room. The measurement was taken in mm at the distance between temporal orbital rims, the deepest palpable point of the angle, and the apex of the cornea as described by Park et al. previously^11^. Right eye readings were taken before left eye readings without removing the instrument from the orbital rims. Exophthalmometry was assessed using a one-mirror Hertel exophthalmometer (Hertel Exophthalmometer, Oculus, Wetzlar, Germany).

The cross-sectional imaging study closest in time to documented Hertel-exophthalmometry was retrieved for each patient from the institutional picture-archiving-and-communication system (PACS, Syngo, Siemens Healthineers, Erlangen, Germany) and displayed on 21-inch 5K-monitors licensed for medical image interpretation for consensus review in random order by one radiology attending with an interest in head-and-neck radiology and more than 20 years of post-fellowship experience, and one Ophthalmologist specializing in oculoplastic surgery. Both observers were blinded to the patients’ final diagnosis and had no access to other clinical or imaging information.

Diagnostic multi-detector-row CT scans were performed at 120 KVp with a primary in-plane resolution of 0.4 mm, based on a 200 mm field-of-view and a 512-point matrix. Intravenous contrast media in standard doses were administered for imaging in the venous phase, approximately 60 s after commencing intravenous injection of contrast media, followed by normal saline solution, in 91 patients (unenhanced CT was performed for bony assessment prior to orbital decompression in suspected dysthyroid optic neuropathy in 20 patients and in 2 patients who reported previous severe reaction to intravenous contrast media).

Secondary multiplanar CT-image-reformatting included 120 mm-fields-of-view, resulting in a nominal in-plane resolution of 0.23 mm, which was adapted to the morphological dimensions of the orbit. Through-plane slice thicknesses were between 0.6 mm and 3.0 mm, respectively.

**Figure 1:**
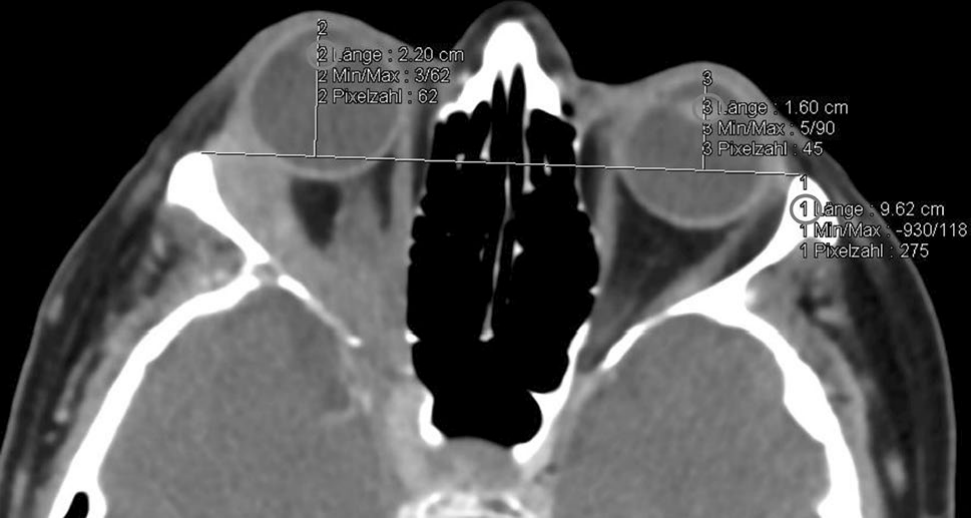
Exemplary CT measurement in the axial plane bisecting the lens: Distance between the lateral orbital rims (1) and perpendicular distance to the corneal apex (2) and (3) in a patient with 6 mm proptosis of the right eye due to adenoidcystic carcinoma of the lacrimal gland with deep orbital invasion (CT with contrast agent, soft tissue window).

Measurement of exophthalmos was taken from both lateral orbital rims to the corneal surface in the axial plane on the section that bisects the lens and recorded in mm (for exemplary measurement see Fig. 1).

Submillimeter measurements were rounded mathematically. Images were reviewed in the soft tissue and bone window.

For further analysis, the final orbital diagnoses were grouped into neoplastic (benign = group 1, malignant = group 2), inflammatory (group 3) and miscellaneous conditions (group 4).

Statistical data collection was performed using Microsoft Excel (Microsoft Corporation, Redmond, WA, USA) for Mac 2011 and analysis was performed with SPSS 25.0 (IBM Corporation, Armonk, NY, USA). Descriptive statistical analyses, Bland–Altman plots, Pearson’s coefficient, Kruskal–Wallis analysis and Mann–Whitney-U-test were employed for statistical analysis. *p* < 0.05 was considered statistically significant.

### Ethics approval and consent to participate

The ethics-committee of Ludwig-Maximilians-University, Munich, Germany, waived ethical approval and individual patient consent for this retrospective single-center observational cohort-study in selected patients (vote number 20-633-KB). However, written informed consent for MDCT-work-up was obtained from all patients. All patient data is non-identifiable and complied with relevant data protection and privacy regulation. All methods were performed in accordance with the relevant guidelines and regulations.

## Results

We included 46 (40.7%) male and 67 (59.3%) female patients into our analysis. Patients’ median age was 57.8 (mean 56.8, range 15.8–90.2, SD ± 16.6) years.

**Figure 2:**
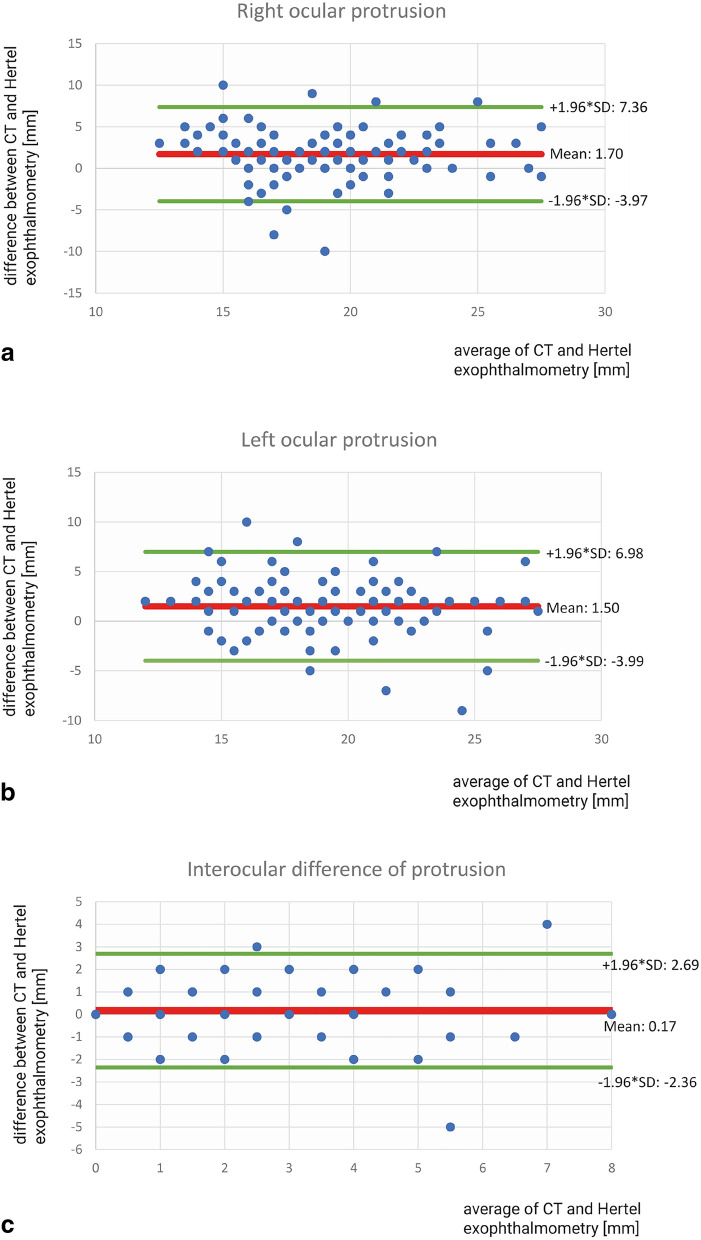
(**a**–**c**) Respective Bland–Altman plots of Hertel-exophthalmometry and CT measurements agreement: Mean difference (red horizontal line) and upper and lower 95% confidence intervals (green horizontal lines) on right ocular protrusion (**a**), left ocular protrusion (**b**) as well as on interocular difference of protrusion (**c**) in 113 patients.

The difference of proptosis in Hertel exophthalmometry was 0–2 mm in 69 (61.1%), and > 2 mm in 42 (38.9%) of patients (CT measurements 0–2 mm in 64 (56.6%), and > 2 mm in 49 (43.4%) of patients, respectively). Mean values for the base of the measurement (distance between the orbital rims) were 97.9 (SD ± 4.0) mm in CT and 113.7 (SD ± 4.9) mm in Hertel exophthalmometry. Consequently, mean clinical Hertel measurements of the base were 15.8 mm wider. Mean values of proptosis were 19.9 (SD ± 3.5) mm in CT and 18.2 (SD ± 3.5) mm in Hertel measurements for the right and 20.1 (SD ± 3.6) mm in CT and 18.6 (SD ± 3.9) mm in Hertel exophthalmometry for the left eye (for respective Bland–Altman plots see Fig. 2a,b).

The mean difference of proptosis between both eyes was 2.4 (SD ± 2.0) mm in CT evaluation and 2.2 (SD ± 2.0) mm in Hertel exophthalmometry (for respective Bland–Altman plot see Fig. 2c).

Pearson’s coefficient (r) was 0.429 when comparing the measurements of the base between CT and Hertel findings and 0.793 when comparing proptosis with both methods (both *p* < 0.001, respectively). In subgroup analysis of patients with a proptosis of 0–2 mm and > 2 mm via CT assessment, Pearson’s coefficient (r) was 0.434 and 0.619 (*p* < 0.001, respectively) proving a higher correlation in patients with higher grade proptosis.

**Figure 3:**
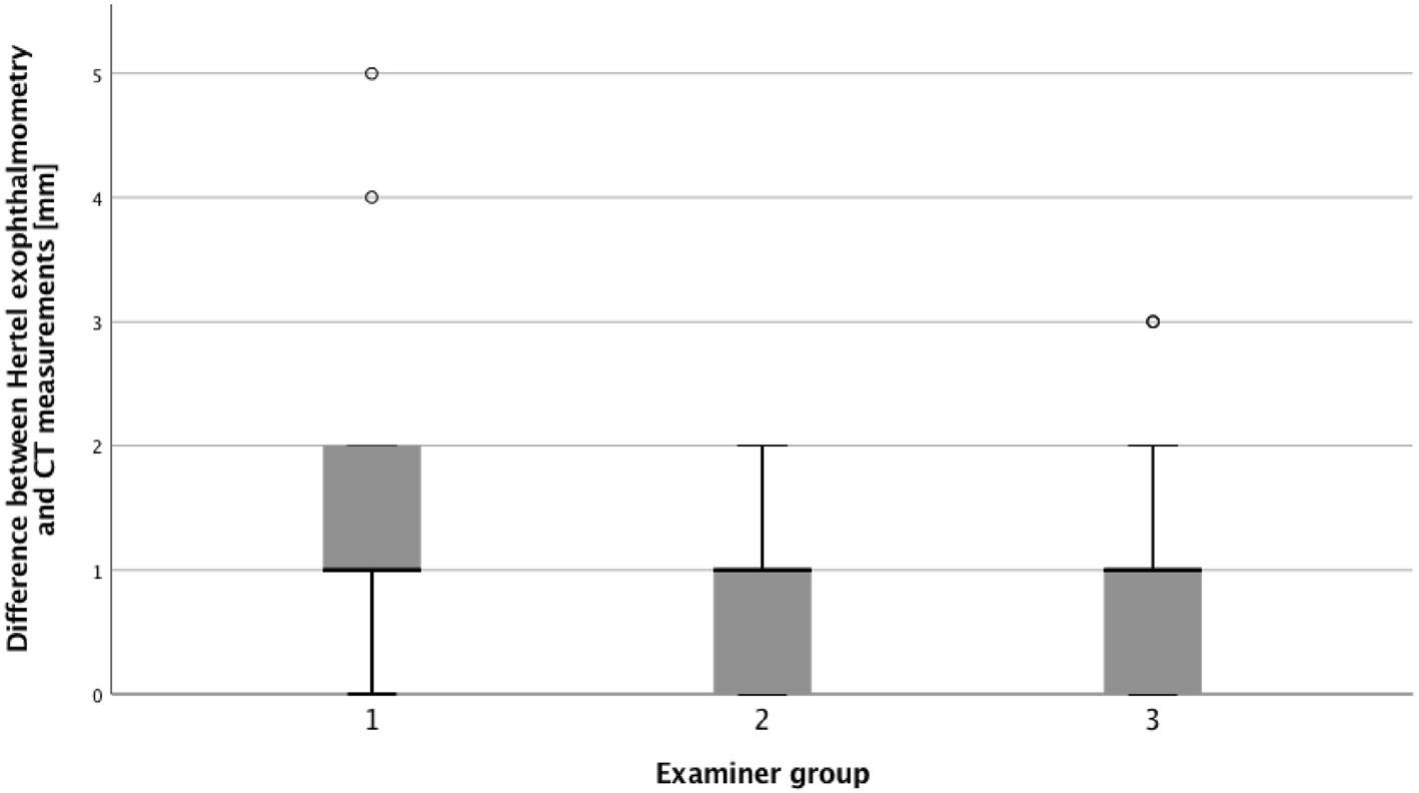
Box-plot showing inter-observer variability in proptosis measurements via Hertel exophthalmometry (< 5 years (group 1), 5–15 years (group 2) and > 25 years (group 3) of clinical experience).

There was a significant difference between the exactness of clinical proptosis measurement when comparing the groups of examiners (< 5 years (n = 31), 5–15 years (n = 24) and > 25 years of clinical experience (n = 58), *p* = 0.042, Kruskal–Wallis analysis). More specifically, precision of Hertel exophthalmometry was dependent from years of the examiners’ expertise when compared with the reproducible and permanent CT measurements (*p* = 0.086 group 1 vs. 2, *p* = 0.014 group 1 vs. 3, *p* = 0.688 group 2 vs. 3, Mann–Whitney-U-test) (Fig. 3).

Subsequently, Pearson’s coefficient (r) was lower for examiner group 1 than groups 2 and 3 (0.691 versus 0.837 and 0.831, respectively, *p* = 0.01).

Proptosis values were not statistically different when grouped by disease entities (benign (n = 25) and malignant neoplasms (n = 35), orbital inflammation (n = 45) and miscellaneous conditions (n = 8), Kruskal–Wallis analysis). There was a trend showing lower values of proptosis solely for the smallest subgroup of miscellaneous conditions (group 4; including pseudoexophthalmos as diagnosis of exclusion).

**Figure 4:**
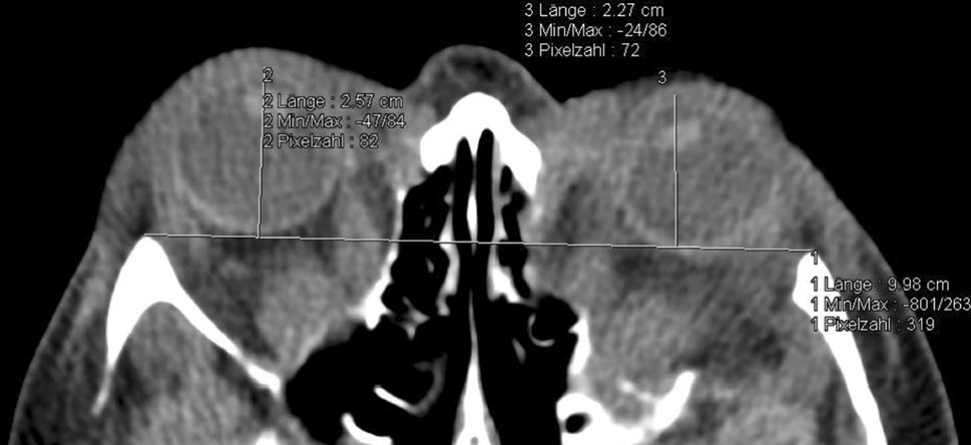
Possible challenges in axial CT measurement of proptosis: Bias of drawing the perpendicular line from the baseline connecting both orbital rims to the corneal apex in a patient with apparent esotropia of the left eye in a patient suffering from dysthyroid optic neuropathy (axial CT with contrast agent, soft tissue window).

**Figure 5:**
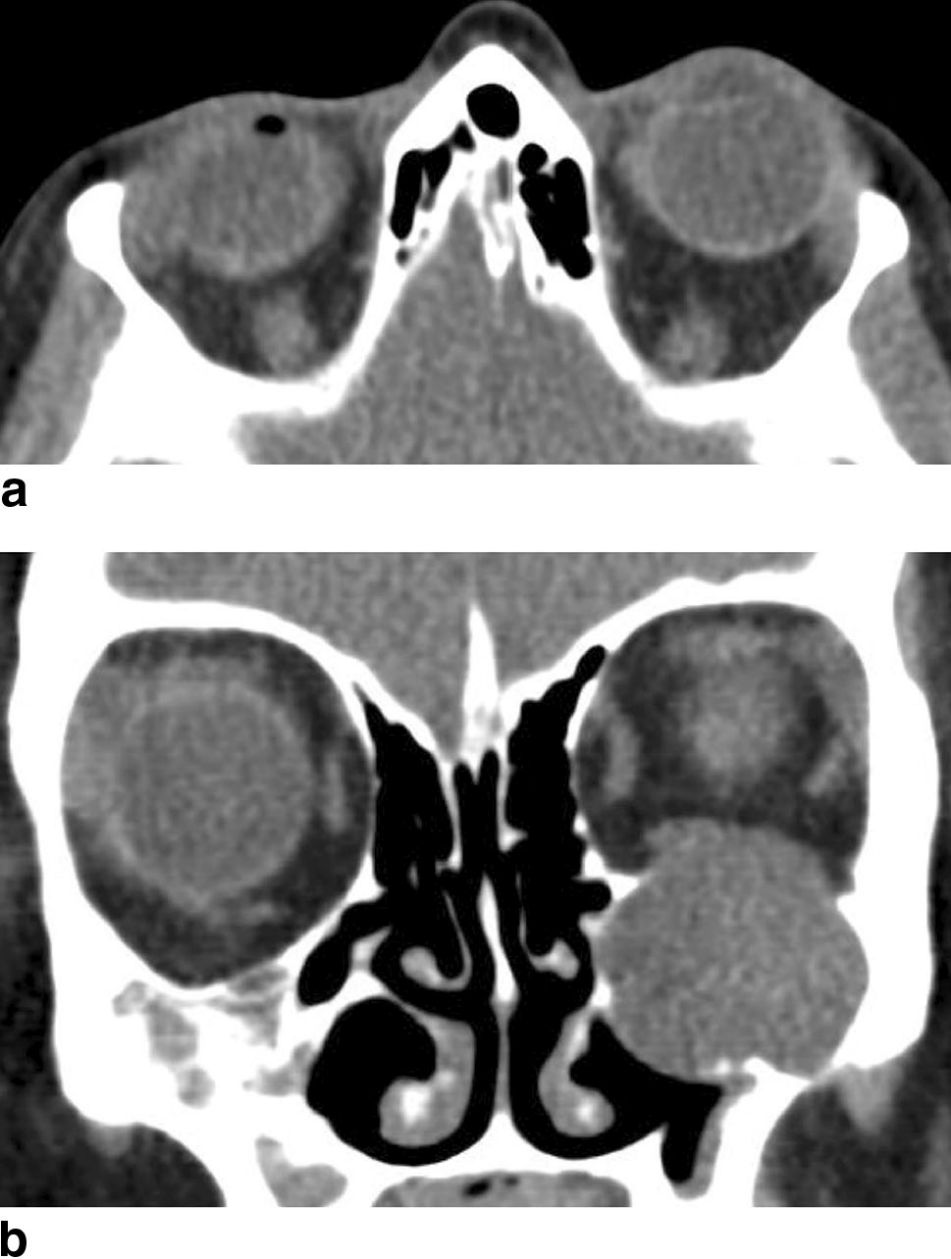
(**a**,**b**) Possible challenges in axial CT measurement of proptosis: Bias caused by no section available bisecting both lenses due to apparent vertical displacement of the left globe resulting from a mucocele of the maxillary sinus [(**a**) axial and (**b**) coronal CT with contrast agent, soft tissue window].

The most challenging proptosis measurements (on CT as well as clinically) included patients with apparent strabismus (Fig. 4) and vertical globe dislocation (Fig. 5a,b).

## Discussion

Exophthalmos is a frequent symptom in orbital pathologies, oftentimes resulting in diagnostic cross-sectional imaging for biopsy- or therapy-planning^6^. In a recent study, Park et al. could prove the best correlation of CT assessment with Hertel exophthalmometry with the method applied in the present study^11^. With this method, Pearson’s coefficients similar or even higher compared to our study could be obtained (r = 0.793 in our study; r = 0.727, Park et al. and r = 0.93, Ramli et al.)^11,14^. Thus, inclusion of axial CT measurement of proptosis taken from both lateral orbital rims to the corneal surface as described above for standardized evaluation of orbital pathologies seems reasonable. Park et al. found less correlation (lower Pearson’s coefficient for patients with a difference in proptosis of ≥ 2 mm) for this CT method^11^. When dividing our patient collective into subgroups for analysis (0–2 mm (we took this cut-off because a difference up to 2 mm can be considered as anatomic variance) and > 2 mm proptosis), we found a higher Pearson’s correlation of 0.619 for proptosis values of > 2 mm. Thus, this CT method has proven feasible for proptosis measurement of orbital pathologies.

For precise radiographic CT evaluation, correct positioning of the patient is mandatory to obtain the section depicting both lenses on the axial plane correctly. If both eyes are not located on the axial plane on CT imaging due to a patient’s head tilt or vertical eye displacement, significant errors may occur^11^. This is especially challenging in patients that suffer from vertical displacement of the globe. Yet, for clinical assessment, the Hertel exophthalmometer must also be tilted in these cases, possibly resulting in a lack of precision as well. This limitation could be largely overcome on CT by choosing the smallest slice-thickness available. Higher resolution would result in more than one slice bisecting the lens and the radiologist could thus perform proptosis measurement on the axial slice most suitable. Patients who require the smallest slice-size could be predetermined on the request of the clinician sending the patient for cross-sectional imaging. This of course implies consequent interdisciplinary patient management.

Furthermore, bias on radiographic proptosis measurement as described could result from apparent strabismus (which is not an uncommon condition in e.g. Grave’s orbitopathy) because the perpendicular line through the baseline does not project on the corneal apex. In these cases, for clinical measurement, the patient could be asked to fixate with the alternate eye to avoid this bias. Yet, on radiographic imaging, even if proptosis assessment were performed in an additional plane as proposed by Park et al.^11^ this bias would still persist. To improve this bias, the axial length of the globe measured in the section bisecting the lens and could then be inserted perpendicularly to the baseline starting at the posterior pole. We see potential for further studies in these cases. It has also been proposed previously to consider the center of the globe as an important reference in strabismus patients for evaluation of proptosis on CT^15^.

Additionally, the reliability of CT measurements could be improved in future analyses by 3-dimensional (3D) measurement when comparing 2-dimensional to 3D CT imaging with 3D imaging proving to be significantly more consistent with Hertel exophthalmometry^15^.

For Hertel exophthalmometry, great inter- and intra-observer variability has been noted^4,7,8^. Accordingly, we could confirm that correlation of Hertel values with CT measurements (supposing a higher repeatability and reproducibility)^11^ depended significantly on the clinical experience of the examiner group. Pearson’s correlation was stronger in the examiner groups with > 5 years of clinical experience. Greater focus should be laid on teaching of residents in Ophthalmology on applying the Hertel exophthalmometer accurately. Additionally, by the structured CT evaluation described, this bias could be overcome allowing residents in Radiology to perform high-quality measurements routinely.

Inference from this study is limited in the following ways: First, although only the respective cross-sectional CT imaging examinations closest in time to clinical Hertel measurements were reviewed, due to the retrospective study design, median time between Hertel exophthalmometry and cross-sectional CT-imaging was 4 days. Yet, as there was no patient with acute retrobulbar hemorrhage or surgery in the meantime, we presume that this time difference should not be relevant. Second, also due to the retrospective nature of the study, the subgroups of clinical examiners and diagnoses included differed in size, but again, this was overcome by non-parametric testing. Third, in retrospect, inter-observer reliability could only be evaluated by comparing correlation of Hertel values to CT assessment and not by all groups of examiners performing their measurements on the same patient.

## Conclusion

Overall, standardized radiographic assessment of exophthalmos correlated well with Hertel exophthalmometry. In Ophthalmology, focus should be laid on accurate teaching of Hertel exophthalmometry, as accuracy of Hertel measurements depended on the experience of the examiner. Furthermore, standardized measurement of proptosis should be performed routinely on available radiographic evaluation of orbital pathologies. Permanent record of these images is an advantage in comparison to clinical assessment alone, especially regarding follow-up examinations.

## Data Availability

The blinded datasets used and analyzed during the current study are available from the corresponding author upon reasonable request.
